# The clinical and genetic spectrum of autosomal-recessive *TOR1A*-related disorders

**DOI:** 10.1093/brain/awad039

**Published:** 2023-02-09

**Authors:** Afshin Saffari, Tracy Lau, Homa Tajsharghi, Ehsan Ghayoor Karimiani, Ariana Kariminejad, Stephanie Efthymiou, Giovanni Zifarelli, Tipu Sultan, Mehran Beiraghi Toosi, Sahar Sedighzadeh, Victoria Mok Siu, Juan Darío Ortigoza-Escobar, Aisha M AlShamsi, Shahnaz Ibrahim, Nouriya Abbas Al-Sannaa, Walla Al-Hertani, Whalen Sandra, Mark Tarnopolsky, Shahryar Alavi, Chumei Li, Debra-Lynn Day-Salvatore, Maria Jesús Martínez-González, Kristin M Levandoski, Emma Bedoukian, Suneeta Madan-Khetarpal, Michaela J Idleburg, Minal Juliet Menezes, Aishwarya Siddharth, Konrad Platzer, Henry Oppermann, Martin Smitka, Felicity Collins, Monkol Lek, Mohmmad Shahrooei, Maryam Ghavideldarestani, Isabella Herman, John Rendu, Julien Faure, Janice Baker, Vikas Bhambhani, Laurel Calderwood, Javad Akhondian, Shima Imannezhad, Hanieh Sadat Mirzadeh, Narges Hashemi, Mohammad Doosti, Mojtaba Safi, Najmeh Ahangari, Paria Najarzadeh Torbati, Soheila Abedini, Vincenzo Salpietro, Elif Yilmaz Gulec, Safieh Eshaghian, Mohammadreza Ghazavi, Michael T Pascher, Marina Vogel, Angela Abicht, Sébastien Moutton, Ange-Line Bruel, Claudine Rieubland, Sabina Gallati, Tim M Strom, Hanns Lochmüller, Mohammad Hasan Mohammadi, Javeria Raza Alvi, Elaine H Zackai, Beth A Keena, Cara M Skraban, Seth I Berger, Erin H Andrew, Elham Rahimian, Michelle M Morrow, Ingrid M Wentzensen, Francisca Millan, Lindsay B Henderson, Hormos Salimi Dafsari, Heinz Jungbluth, Natalia Gomez-Ospina, Anne McRae, Merlene Peter, Danai Veltra, Nikolaos M Marinakis, Christalena Sofocleous, Farah Ashrafzadeh, Davut Pehlivan, Johannes R Lemke, Judith Melki, Audrey Benezit, Peter Bauer, Denisa Weis, James R Lupski, Jan Senderek, John Christodoulou, Wendy K Chung, Rose Goodchild, Amaka C Offiah, Andres Moreno-De-Luca, Mohnish Suri, Darius Ebrahimi-Fakhari, Henry Houlden, Reza Maroofian

**Affiliations:** Department of Neurology, Boston Children's Hospital, Harvard Medical School, Boston, MA, USA; Division of Child Neurology and Inherited Metabolic Diseases, Heidelberg University Hospital, Heidelberg, Germany; Department of Neuromuscular Diseases, Queen Square Institute of Neurology, University College London, London, UK; School of Health Sciences, Division of Biomedicine, University of Skovde, Skovde, Sweden; Molecular and Clinical Sciences Institute, St. George's, University of London, Cranmer Terrace, London, UK; Department of Medical Genetics, Next Generation Genetic Polyclinic, Mashhad, Iran; Kariminejad-Najmabadi Pathology & Genetics Center, Tehran, Iran; Department of Neuromuscular Diseases, Queen Square Institute of Neurology, University College London, London, UK; CENTOGENE GmbH, Am Strande 7, 18055 Rostock, Germany; Department of Neuromuscular Diseases, Queen Square Institute of Neurology, University College London, London, UK; Department of Pediatrics, School of Medicine, Mashhad University of Medical Sciences, Mashhad, Iran; Neuroscience Research Center, Mashhad University of Medical Sciences, Mashhad, Iran; Department of Biological Sciences, Faculty of Science, Shahid Chamran University of Ahvaz, Ahvaz, Iran; KaryoGen, Isfahan, Iran; Division of Medical Genetics, Department of Pediatrics, Schulich School of Medicine and Dentistry, Western University, London, ON, Canada; Movement Disorders Unit, Pediatric Neurology Department, Institut de Recerca, Hospital Sant Joan de Déu Barcelona, Barcelona, Spain; Genetic Division, Pediatrics Department, Tawam Hospital, Al Ain, UAE; Department of pediatrics and child Health, Aga Khan University, Karachi, Pakistan; Pediatric Services, John Hopkins Aramco Health Care, Dhahran, Saudi Arabia; Harvard Medical School, Boston Children's Hospital, Department of Pediatrics, Division of Genetics and Genomics, Boston, MA, USA; APHP UF de Génétique Clinique, Centre de Référence des Anomalies du Développement et Syndromes Malformatifs, APHP, Hôpital Armand Trousseau, ERN ITHACA, Sorbonne Université, Paris, France; Department of Pediatrics (MT – Neuromuscular and Neurometabolics, CL – Medical Genetics), McMaster Children's Hospital, Hamilton, Ontario, Canada; Department of Neuromuscular Diseases, Queen Square Institute of Neurology, University College London, London, UK; Department of Pediatrics (MT – Neuromuscular and Neurometabolics, CL – Medical Genetics), McMaster Children's Hospital, Hamilton, Ontario, Canada; The Department of Medical Genetics and Genomic Medicine at Saint Peter's University Hospital, New Brunswick, NJ, USA; Pediatric Neurology Unit, Cruces University Hospital, Barakaldo, Vizcaya, Spain; The Department of Medical Genetics and Genomic Medicine at Saint Peter's University Hospital, New Brunswick, NJ, USA; Roberts Individualized Medical Genetics Center, Children's Hospital of Philadelphia, Philadelphia, PA, USA; Division of Genetic and Genomic Medicine, Department of Pediatrics, UPMC Children's Hospital of Pittsburgh, Pittsburgh, Pennsylvania, USA; Division of Genetic and Genomic Medicine, Department of Pediatrics, UPMC Children's Hospital of Pittsburgh, Pittsburgh, Pennsylvania, USA; Department of Anaesthesia, the Children's Hospital at Westmead, Sydney, NSW, Australia; Discipline of Child and Adolescent Health, and Specialty of Genomic Medicine, Sydney Medical School, Sydney University, Sydney, NSW, Australia; Harvard Medical School, Boston Children's Hospital, Department of Pediatrics, Division of Genetics and Genomics, Boston, MA, USA; Institute of Human Genetics, University of Leipzig Medical Center, Leipzig, Germany; Institute of Human Genetics, University of Leipzig Medical Center, Leipzig, Germany; Department of Neuropediatrics, Medical Faculty Carl Gustav Carus, Technical University Dresden, Dresden, Germany; Discipline of Child and Adolescent Health, and Specialty of Genomic Medicine, Sydney Medical School, Sydney University, Sydney, NSW, Australia; Department of Clinical Genetics, Children's Hospital at Westmead, Sydney, NSW, Australia; Department of Genetics, Yale School of Medicine, New Haven, Connecticut, USA; Medical Laboratory of Dr. Shahrooei, Tehran, Iran; Department of Microbiology and Immunology, Clinical and Diagnostic Immunology, KU Leuven, Leuven, Belgium; Medical Laboratory of Dr. Shahrooei, Tehran, Iran; Section of Pediatric Neurology and Developmental Neuroscience, Department of Pediatrics, Baylor College of Medicine, Houston, TX, USA; Department of Molecular and Human Genetics, Baylor College of Medicine, Houston, TX, USA; Texas Children's Hospital, Houston, TX, USA; Division of Pediatric Neuroscience, Boys Town National Research Hospital, Boys Town, NE, USA; Univ. Grenoble Alpes, Inserm, U1216, CHU Grenoble Alpes, Grenoble Institut Neurosciences, Grenoble, France; Univ. Grenoble Alpes, Inserm, U1216, CHU Grenoble Alpes, Grenoble Institut Neurosciences, Grenoble, France; Division of Genetics and Genomic Medicine, Children's Hospital and Clinics of Minnesota, Minneapolis, Minnesota, USA; Division of Genetics and Genomic Medicine, Children's Hospital and Clinics of Minnesota, Minneapolis, Minnesota, USA; Lucile Packard Children's Hospital Stanford, Palo Alto, CA, USA; Department of Pediatrics, Division of Medical Genetics, Stanford University School of Medicine, Stanford, CA, USA; Pediatric Neurology Department, Ghaem Hospital, Mashhad University of Medical Sciences, Mashhad, Iran; Department of Pediatric Neurology, Faculty of Medicine, Mashhad University of Medical Sciences, Mashhad, Iran; Department of Pediatric Neurology, Faculty of Medicine, Mashhad University of Medical Sciences, Mashhad, Iran; Department of Pediatrics, School of Medicine, Mashhad University of Medical Sciences, Mashhad, Iran; Department of Medical Genetics, Next Generation Genetic Polyclinic, Mashhad, Iran; Department of Medical Genetics, Next Generation Genetic Polyclinic, Mashhad, Iran; Innovative medical research centre, Mashhad branch, Islamic Azad University, Mashhad, Iran; Department of Medical Genetics, Next Generation Genetic Polyclinic, Mashhad, Iran; Department of Neuromuscular Diseases, Queen Square Institute of Neurology, University College London, London, UK; Department of Neuromuscular Diseases, Queen Square Institute of Neurology, University College London, London, UK; Istanbul Medeniyet University Medical School, Department of Medical Genetics, Istanbul, Turkey; Isfahan Fertility and Infertility Center, Isfahan, Iran; Department of Pediatric Neurology, Imam Hossein Children's Hospital, Isfahan University of Medical Sciences, Isfahan, Iran; Friedrich-Baur-Institute at the Department of Neurology, University Hospital, LMU Munich, Munich, Germany; Friedrich-Baur-Institute at the Department of Neurology, University Hospital, LMU Munich, Munich, Germany; Deutsches Krebsforschungszentrum, Heidelberg, Germany; Friedrich-Baur-Institute at the Department of Neurology, University Hospital, LMU Munich, Munich, Germany; Medizinisch Genetisches Zentrum, Munich, German; Multidisciplinary Center for Prenatal Diagnosis, Pôle Mère Enfant, Maison de Santé Protestante Bordeaux Bagatelle, Talence, France; Équipe Génétique des Anomalies du Développement (GAD), INSERM UMR1231, Dijon, France; Unité Fonctionnelle Innovation en Diagnostic génomique des maladies rares, FHU-TRANSLAD, Dijon University Hospital, Dijon, France; Division of Human Genetics, Department of Pediatrics, Inselspital, University of Bern, Switzerland; Division of Human Genetics, Department of Pediatrics, Inselspital, University of Bern, Switzerland; Institute of Human Genetics, Klinikum rechts der Isar, Technical University Munich, Munich, Germany; Children's Hospital of Eastern Ontario Research Institute, University of Ottawa, Ottawa, Canada; Division of Neurology, Department of Medicine, The Ottawa Hospital, Ottawa, Canada; Department of pediatrics, Zabol University of medical sciences, Zabol, Iran; Department of Pediatric Neurology, The Children's Hospital and the University of Child Health Sciences, Lahore, Pakistan; Division of Human Genetics, Children's Hospital of Philadelphia, University of Pennsylvania School of Medicine, Philadelphia, PA, USA; Division of Human Genetics, Children's Hospital of Philadelphia, University of Pennsylvania School of Medicine, Philadelphia, PA, USA; Division of Human Genetics, Children's Hospital of Philadelphia, University of Pennsylvania School of Medicine, Philadelphia, PA, USA; Children's National Research Institute, Washington DC, USA; Children's National Research Institute, Washington DC, USA; Haghighat Medical Imaging center–Tehran, Tehran, Iran; GeneDx, Gaithersburg, MD, USA; GeneDx, Gaithersburg, MD, USA; GeneDx, Gaithersburg, MD, USA; GeneDx, Gaithersburg, MD, USA; Department of Pediatrics, Faculty of Medicine and University Hospital Cologne, University of Cologne, Cologne, Germany; Max-Planck-Institute for Biology of Ageing and CECAD, Cologne, Germany; Department of Paediatric Neurology - Neuromuscular Service, Evelina London Children's Hospital, Guy's & St Thomas' Hospital NHS Foundation Trust, London, UK; Department of Paediatric Neurology - Neuromuscular Service, Evelina London Children's Hospital, Guy's & St Thomas' Hospital NHS Foundation Trust, London, UK; Randall Centre for Cell and Molecular Biophysics, Muscle Signalling Section, Faculty of Life Sciences and Medicine (FoLSM), King's College London, London, UK; Department of Pediatrics, Stanford University, Stanford, CA, USA; Division of Genetics, Genomics, and Metabolism, Ann and Robert H. Lurie Children's Hospital of Chicago, Chicago, USA; Division of Genetics, Genomics, and Metabolism, Ann and Robert H. Lurie Children's Hospital of Chicago, Chicago, USA; Laboratory of Medical Genetics, Medical School, National and Kapodistrian University of Athens, St. Sophia's Children's Hospital, Athens, Greece; Laboratory of Medical Genetics, Medical School, National and Kapodistrian University of Athens, St. Sophia's Children's Hospital, Athens, Greece; Laboratory of Medical Genetics, Medical School, National and Kapodistrian University of Athens, St. Sophia's Children's Hospital, Athens, Greece; Department of Pediatric Neurology, Faculty of Medicine, Mashhad University of Medical Sciences, Mashhad, Iran; Section of Pediatric Neurology and Developmental Neuroscience, Department of Pediatrics, Baylor College of Medicine, Houston, TX, USA; Department of Molecular and Human Genetics, Baylor College of Medicine, Houston, TX, USA; Texas Children's Hospital, Houston, TX, USA; Institute of Human Genetics, University of Leipzig Medical Center, Leipzig, Germany; Center for Rare Diseases, University of Leipzig Medical Center, Leipzig, Germany; Institut National de la Santé et de la Recherche Médicale (Inserm), UMR-1195, Université Paris Saclay, Le Kremlin Bicêtre, 94276, Paris, France; Neurologie et réanimation pédiatrique, Hôpital Raymond Poincaré, APHP, Garches, France; CENTOGENE GmbH, Am Strande 7, 18055 Rostock, Germany; Department of Medical Genetics, Kepler University Hospital, Johann Kepler University, Linz, Austria; Department of Molecular and Human Genetics, Baylor College of Medicine, Houston, TX, USA; Texas Children's Hospital, Houston, TX, USA; Human Genome Sequencing Center, Baylor College of Medicine, Houston, TX, USA; Department of Pediatrics, Baylor College of Medicine, Houston, TX, USA; Friedrich-Baur-Institute at the Department of Neurology, University Hospital, LMU Munich, Munich, Germany; Discipline of Child and Adolescent Health, and Specialty of Genomic Medicine, Sydney Medical School, Sydney University, Sydney, NSW, Australia; Murdoch Children's Research Institute, Melbourne and Department of Paediatrics, Melbourne Medical School, University of Melbourne, Melbourne, VIC, Australia; Department of Pediatrics and Medicine, Columbia University New York, NY, USA; KU Leuven Department of Neurosciences, Leuven Brain Institute, Leuven, Belgium; VIB-KU Leuven Center for Brain and Disease Research, Laboratory for Dystonia Research, Leuven, Belgium; Department of Oncology & Metabolism, University of Sheffield, UK; Autism & Developmental Medicine Institute, Genomic Medicine Institute, Department of Radiology, Diagnostic Medicine Institute, Geisinger, Danville, PA, USA; Clinical Genetics Service, Nottingham University Hospitals NHS Trust, Nottingham, UK; Department of Neurology, Boston Children's Hospital, Harvard Medical School, Boston, MA, USA; Movement Disorders Program, Department of Neurology, Boston Children's Hospital, Harvard Medical School, Boston, MA, USA; The Manton Center for Orphan Disease Research, Boston Children's Hospital, Boston, MA, USA; Intellectual and Developmental Disabilities Research Center, Boston Children's Hospital, Boston, MA, USA; Department of Neuromuscular Diseases, Queen Square Institute of Neurology, University College London, London, UK; Department of Neuromuscular Diseases, Queen Square Institute of Neurology, University College London, London, UK

**Keywords:** AMC5, arthrogryposis multiplex congenita 5, biallelic variation, NDD, Torsin-1A

## Abstract

In the field of rare diseases, progress in molecular diagnostics led to the recognition that variants linked to autosomal-dominant neurodegenerative diseases of later onset can, in the context of biallelic inheritance, cause devastating neurodevelopmental disorders and infantile or childhood-onset neurodegeneration. *TOR1A*-associated arthrogryposis multiplex congenita 5 (AMC5) is a rare neurodevelopmental disorder arising from biallelic variants in *TOR1A*, a gene that in the heterozygous state is associated with torsion dystonia-1 (DYT1 or DYT-*TOR1A*), an early-onset dystonia with reduced penetrance. While 15 individuals with AMC5-*TOR1A* have been reported (less than 10 in detail), a systematic investigation of the full disease-associated spectrum has not been conducted.

Here, we assess the clinical, radiological and molecular characteristics of 57 individuals from 40 families with biallelic variants in *TOR1A*. Median age at last follow-up was 3 years (0–24 years). Most individuals presented with severe congenital flexion contractures (95%) and variable developmental delay (79%). Motor symptoms were reported in 79% and included lower limb spasticity and pyramidal signs, as well as gait disturbances. Facial dysmorphism was an integral part of the phenotype, with key features being a broad/full nasal tip, narrowing of the forehead and full cheeks. Analysis of disease-associated manifestations delineated a phenotypic spectrum ranging from normal cognition and mild gait disturbance to congenital arthrogryposis, global developmental delay, intellectual disability, absent speech and inability to walk. In a subset, the presentation was consistent with foetal akinesia deformation sequence with severe intrauterine abnormalities. Survival was 71%, with higher mortality in males. Death occurred at a median age of 1.2 months (1 week–9 years), due to respiratory failure, cardiac arrest or sepsis. Analysis of brain MRI studies identified non-specific neuroimaging features, including a hypoplastic corpus callosum (72%), foci of signal abnormality in the subcortical and periventricular white matter (55%), diffuse white matter volume loss (45%), mega cisterna magna (36%) and arachnoid cysts (27%). The molecular spectrum included 22 distinct variants, defining a mutational hotspot in the C-terminal domain of the Torsin-1A protein. Genotype-phenotype analysis revealed an association of missense variants in the 3-helix bundle domain to an attenuated phenotype, while missense variants near the Walker A/B motif as well as biallelic truncating variants were linked to early death.

In summary, this systematic cross-sectional analysis of a large cohort of individuals with biallelic *TOR1A* variants across a wide age-range delineates the clinical and genetic spectrum of *TOR1A*-related autosomal-recessive disease and highlights potential predictors for disease severity and survival.

## Teaser

In this cross-sectional analysis, Saffari *et al.* systematically assess the clinical, radiological, and molecular characteristics of 56 individuals with autosomal-recessive *TOR1A*-related disease. They delineate a broad and variable phenotypic spectrum, define core clinical symptoms, and highlight predictors for disease severity and survival.

## Introduction

*TOR1A*-associated arthrogryposis multiplex congenita 5 (AMC5, MIM: #618947) is a rare congenital disorder arising from biallelic variants in *TOR1A*. *TOR1A* encodes for Torsin-1A, a member of the AAA+ family of adenosine triphosphatases (ATPases) that localizes to the endoplasmic reticulum where it has been implicated in a variety of cellular functions, including protein folding, lipid metabolism, cytoskeletal organization and nuclear polarity.^1-4^ While heterozygous variant alleles in *TOR1A* cause classic early-onset torsion dystonia^5,6^ (DYT1 or DYT-*TOR1A*,^7^ MIM: #128100), patients with biallelic variants present with a distinct phenotype characterized by severe congenital arthrogryposis and a complex neurodevelopmental disorder (NDD).^8-10^ To date, 15 individuals with AMC5-*TOR1A* have been reported (less than 10 in detail). A systematic investigation of the full clinical, radiological, and molecular spectrum, including exploration of genotype-phenotype correlations, has not been conducted.

Here, we systematically analyse a cohort of 57 individuals from 40 families with biallelic *TOR1A* variants, including all previously published cases.^8-17^ We show that biallelic *TOR1A* variants cause a broad phenotypic spectrum ranging from mildly affected individuals with minimal motor impairment and normal cognition to severe cases with congenital arthrogryposis, a syndromic NDD and early death. We further identify potential genetic predictors for disease severity and mortality.

## Materials and methods

### Patient ascertainment

**Figure 1:**
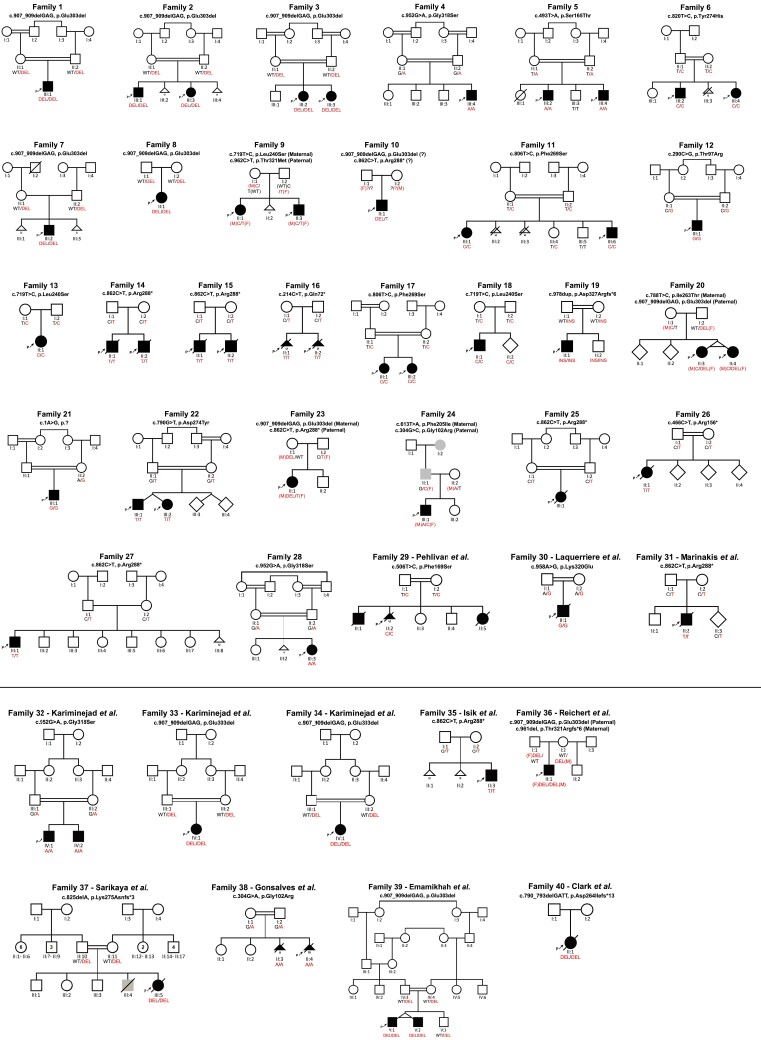
**Pedigrees and segregation results.** Individuals with *TOR1A*-related disorders are indicated by filled black shapes with an arrow pointing to the proband. Open shapes represent unaffected individuals. Diagonal lines across indicate deceased individuals. Squares represent males, circles represent females, diamonds indicate unknown gender, triangles without a diagonal line represent miscarriages, while triangles with a diagonal line represents termination of pregnancy. Grey shaded shapes indicate individuals with hereditary spastic paraplegia. Two interconnected lines for II:3 and II:4 in Family 20 as well as V:1 and V:2 in Family 39 indicate monozygotic twins. The presence of consanguinity between two individuals is represented by double lines. Below each individual, A = Generation number; B = Number of that individual in that generation. Segregation results for all individuals tested are indicated with either red (presence of the *TOR1A* variant) and/or black (presence of the reference allele). RED/RED text indicates the presence of *TOR1A* variants in a homozygous or compound heterozygous state, while RED/BLACK text indicates the presence of the *TOR1A* variant in a heterozygous state. The inheritance of the compound heterozygous *TOR1A* variants for Families 9, 10, 20, 23, 24 and 36 are indicated by maternal allele (M), paternal allele (F) or unknown inheritance (?). For previously reported families, a reference to the original report has been included in the family identifier. Families 29–31 have been only briefly reported as part of large heterogenous cohorts with suspected genetic aetiologies. Clinical data for those families have been collected for this study. In Family 29, only individual II:2 has been previously reported. For previously reported families, 32–40, follow-up data have been added where available.

Forty-two individuals with biallelic *TOR1A* variants from 29 independent families were identified through international collaboration, data mining of DNA sequences of multiple diagnostic and research laboratories around the world as well as GeneMatcher.^18^ Pedigrees for newly (Families 1–28) and previously reported (Families 29–40) families are provided in Fig. 1. New and follow-up data were collected from previously published cases^9,10,12,16,17^ through direct correspondence with the authors of the original reports. Among these, three families were only briefly described as parts of large heterogeneous cohorts with suspected genetic aetiologies (Families 29–31).^11,16,17^ For those families, detailed clinical data have been collected. For the remaining families, follow up data have been gathered unless the individuals were deceased at the time of the original reports.^8,11,13-15^ This study was approved by the institutional review boards at University College London (#310045/1571740/37/598), LMU Munich (#084/00), Baylor College of Medicine (#H-29697) and the Kariminejad-Najmabadi Pathology and Genetics ethics committee. Informed consent for the publication of clinical and genetic data, including photographs and videos was obtained from all participants.

Facial photographs were reviewed for 18 children and six previously reported cases.^8,9^ Dysmorphic features were described based on terminology recommended in *Elements of Morphology*.^19^ Where no term was available for a dysmorphic feature, Human Phenotype Ontology (HPO) terminology was used instead. All photographs were reviewed by a board-certified dysmorphologist. Clinically acquired brain MRI studies were available for 11 individuals, including complete DICOM (Digital Imaging and Communications in Medicine) files for four and low-resolution photographs of brain MRIs for seven cases. All imaging studies were reviewed by a board-certified neuroradiologist. Skeletal radiographs were reviewed by a board-certified paediatric radiologist.

### Annotation of Human Phenotype Ontology terms

Phenotypic information was translated into standardized HPO terms (HPO version 1.7.3; release 2020/10/12) as previously described.^20-22^ To allow computation of frequencies in line with commonly used clinical terminology, phenotypic features were regrouped and reassigned to newly generated umbrella terms whenever the original HPO grouping structure seemed clinically inaccurate. For instance, ‘Spasticity HP:0001257’ was reclassified as a subgroup of the HPO term ‘Movement disorder HP:0100022’. Furthermore, the HPO term ‘Stereotypic hand wringing’ was replaced by ‘Stereotypic hand movements’, which more accurately describes motor stereotypies in our cohort. In most cases, assessment of cognitive function was based on clinician assessment rather than formal IQ testing, and individuals were therefore triaged into two groups: ‘Intellectual disability (ID), mild HP:0001256’, for individuals with apparent but minor cognitive deficits and the newly generated term ‘Intellectual disability (ID), moderate to severe HP:0002342, HP:0010864’, pooling cases reported to have ‘moderate’, ‘severe’ or ‘profound’ intellectual disability.

### Molecular testing

Variants in *TOR1A* were identified by certified genetic laboratories and reviewed by board-certified geneticists. All variants were harmonized with the canonical Ensembl feature ENST00000351698.5 (RefSeq NM_000113.3) of the GRCh38/hg38 human reference genome build. A detailed characterization, including allele frequencies and interpretation of the variants using available databases, as well as *in silico* prediction and classification tools is provided in the Supplementary material.

### Haplotype analysis

Haplotype analysis was done by plotting a colour banding of the variants flanking the pathogenic variant for each individual. By comparing the banding patterns of patients and controls, the status of being a founder/recurrent variant was investigated.

### Modeling of torsin-1a protein structure and reported variants

Information on the protein sequence and functional annotation of human Torsin-1A was obtained from the Universal Protein Resource (UniProt) database^23^ (UniProt ID: O14656) and available structural data.^24,25^ Novel intragenic variants identified in this study, as well as previously reported pathogenic variants, were annotated along the Torsin-1A protein structure. To visualize tolerance to genomic variation across the *TOR1A* transcript, Combined Annotation Dependent Depletion (CADD) Phred scores of all possible amino acid substitutions were computed and mapped to the corresponding protein sequence using VarMAP.^26^

### Statistical analysis

Statistical analysis was performed using R version 4.2.0 (2022-04-22) and RStudio (version 2022.02.3 + 492; RStudio, Inc.). Demographic data were summarized descriptively using frequency counts and percentages of the total study population for categorical variables. For continuous variables, the median with the interquartile range (IQR) were used, based on the distribution of data tested by visualization with histograms and quantile-quantile plots, as well as normality testing using the Shapiro–Wilk test. Sample sizes are indicated (*n*) for each analysis.

### Data availability

The data that support the findings of this study are available from the corresponding author, upon reasonable request.

## Results

### Demographic information

A total of 57 individuals from 40 families were included in this study. Fifty-four of fifty-seven cases had genetically confirmed homozygous or compound heterozygous variants in *TOR1A*. In two newly reported individuals (F29-II:1 and F29-II:5) without formal genetic testing, pathogenic variants in *TOR1A* were strongly assumed based on segregation analysis of a previously reported phenotypically similar homozygous sibling and the heterozygous parents (Fig. 1; Family 29^11^). Additionally, in one new family (Family 25), no genetic material was available from the patient (F25-III:1) after death at 8 days of life. A diagnosis of *TOR1A*-related arthrogryposis was made based on the characteristic phenotype and segregation of a recurrent pathogenic *TOR1A* variant in both parents. Where possible, new and follow up-data were collected for previously reported cases.^8-17^ The median age at last follow-up was 3 years (range 0–24). The male-to-female ratio was 1.5:1.

### The phenotypic spectrum of autosomal-recessive *TORA1*-related disease

**Figure 2:**
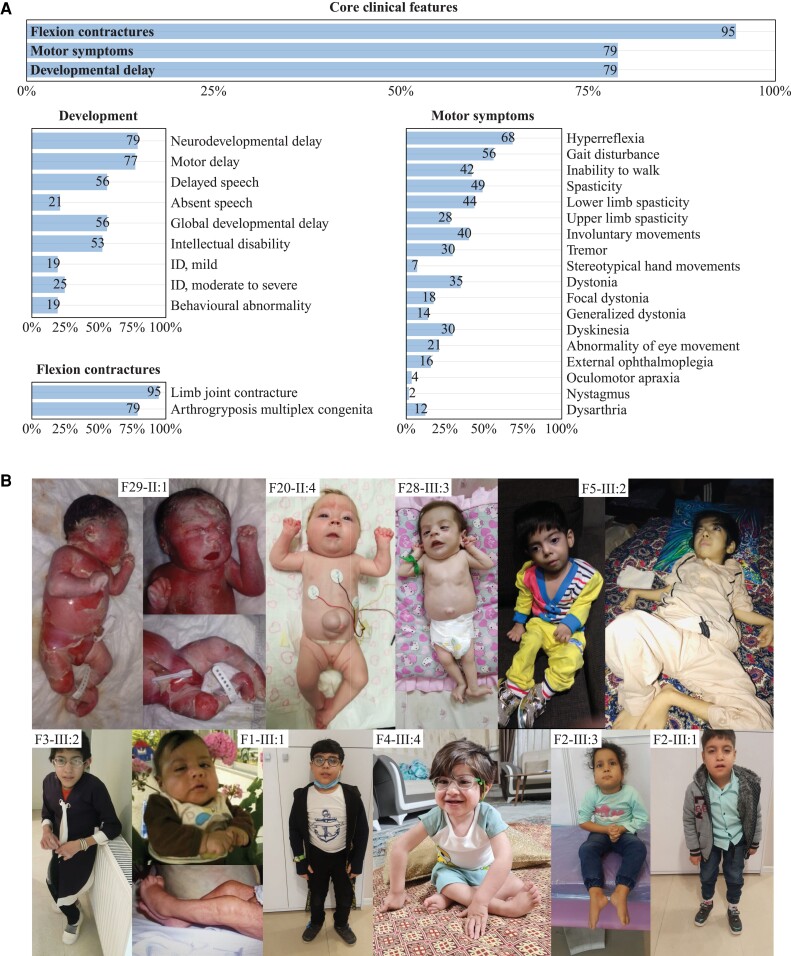
**Clinical spectrum**. (**A**) A total of 57 individuals were analysed. Frequencies of core clinical features were present in the majority of individuals in our cohort. Below, a detailed breakdown of HPO derived phenotypic features for the core symptom categories ‘Development’, ‘Flexion contractures’ and ‘Motor symptoms’ is shown. Frequencies were printed on the respective bars. (**B**) Photographs of individuals with biallelic *TOR1A* variants depicting the spectrum of the disease. (F29-II:1) Foetal akinesia deformation sequence. (F20-II:4 and F28-III:3) Congenital arthrogryposis, umbilical hernia. (F5-III:2) Severe arthrogryposis, minimal motor function. (F3-III:2) Arthrogryposis and scoliosis significantly impacting gait. (F1-III:1) Congenital contractures with improvement in childhood. (F4-III:4) Congenital contractures affecting the lower limbs, right club foot. (F2-III:1) and sibling (F2-III:3) with mild motor impairment, preserved ambulation.

The phenotypic spectrum of core clinical features in our cohort is illustrated in Table 1 and Fig. 2A. A detailed summary of all phenotypic features assessed including stratification for sex is provided in Supplementary Fig. 1 and the Supplementary material. Detailed data for each individual are provided in the Supplementary material.

**Table 1:** Core clinical features of *TOR1A*-related disorders

Clinical features	Frequency
**Pre-/neonatal complications**	
Decreased foetal movement HP:0001558	32% (18/57)
Neonatal respiratory distress HP:0002643	37% (21/57)
Infantile muscular hypotonia HP:0008947	21% (12/57)
**Flexion contracture**	
Limb joint contracture HP:0003121	95% (54/57)
Arthrogryposis multiplex congenita HP:0002804	79% (45/57)
**Development**	
Motor delay HP:0001270	77% (44/57)
Delayed speech HP:0000750	56% (32/57)
Global developmental delay HP:0001263	56% (32/57)
Intellectual disability HP:0001249	53% (30/57)
Behavioural abnormality HP:0000708	19% (11/57)
**Motor symptoms**	
Gait disturbance HP:0001288	56% (32/57)
Inability to walk HP:0002540	42% (24/57)
Movement disorder HP:0100022	75% (43/57)
Hyperreflexia HP:0001347	68% (39/57)
Spasticity HP:0001257	49% (28/57)
Lower limb spasticity HP:0002061	44% (25/57)
Upper limb spasticity HP:0006986	28% (16/57)
Involuntary movements HP:0004305	40% (23/57)
Tremor HP:0001337	30% (17/57)
Stereotypical hand movements HP:0012171	7% (4/57)
Dystonia HP:0001332	35% (20/57)
Focal dystonia HP:0004373	18% (10/57)
Generalized dystonia HP:0007325	14% (8/57)
Dyskinesia HP:0100660	30% (17/57)
Dysarthria HP:0001260	12% (7/57)
**Dysmorphic features** ^a^	
Broad/full nasal tip	95.8% (23/24)
Narrow forehead/bifrontal/bitemporal narrowing	79.2% (19/24)
Full cheeks	70.8% (17/24)
**Neuroradiological features** ^a^	
Hypoplastic corpus callosum	72% (8/11)
Foci of signal abnormality in the subcortical and periventricular white matter	55% (6/11)
Diffuse white matter volume loss	45% (5/11)
Mega cisterna magna	36% (4/11)
Arachnoid cysts	27% (3/11)

Frequencies of dysmorphic and neuroradiological features are based on the total number of cases for which photos/neuroimaging studies available. All other frequencies are based on the entire study population.

Core clinical features that were present in most individuals included flexion contractures (95%), developmental delay (DD) (79%) and motor symptoms (79%) (Table 1 and Fig. 2). Contractures were the most prominent feature in our cohort (*n* = 54/57), involving both upper (*n* = 42) and lower (*n* = 49) limbs, with multiple joints affected in most individuals, consistent with a descriptive diagnosis of arthrogryposis multiplex congenita^27^ (*n* = 45). Additional joint deformities included abnormalities of the spinal curvature such as kyphosis or scoliosis (*n* = 27) as well as defects of hip formation, including congenital hip dislocation (CHD) and developmental dysplasia of the hip (DDH) (*n* = 26). In cases where information on age at onset of contractures was available (*n* = 41), these were detected mostly prenatally or at birth (*n* = 39), and in single cases at postnatal day 10 (F9-II:1), or at 3 years of age (F5-III:2). An exceptionally severe phenotype was reported in three individuals from two families (Families 16 and 29), consistent with a diagnosis of severe foetal akinesia deformation sequence (FADS), a clinically and genetically heterogeneous constellation of features characterized by decreased foetal movement, intrauterine growth restriction, arthrogryposis and developmental anomalies such as lung hypoplasia. Example photographs and a video of patients with flexion contractures at different ages and across the phenotypic spectrum are provided in Fig. 2B and Supplementary Video 1.

Developmental delay with variable severity was present in all cases with available information (79% of individuals) and rated as global developmental delay (GDD) in 56%. ID, present in 53%, was described as mild in 18%, moderate to severe in 25% and not further specified in 10% of individuals. While information on cognitive abilities was unavailable in 33%, in 14% of cases no concerns regarding cognitive impairment were reported. These individuals depicted an attenuated phenotype dominated by contractures (*n* = 7/8), in some cases congenital arthrogryposis (*n* = 5/8), a pyramidal syndrome (*n* = 7/8), preserved ambulation with mild to moderate gait disturbances (*n* = 6/8) and variable speech delay (*n* = 5/8). Overall speech and language development in the cohort was delayed in 56% of individuals with first words spoken at a median age of 21 months (IQR = 22.5). Overall, 21% remained non-verbal at a median age at last follow up of 3 years (range 2.8–18 years). Behavioural problems were reported in 19%, mostly not further specified, but in some cases described as hyperactivity, anxiety and aggressive or impulsive behaviours.

Motor milestones such as unsupported sitting and independent walking were achieved with significant delay at a median age of 17 months (IQR = 24) and 36 months (IQR = 24), respectively. In total, 32% of individuals were never able to walk (median age at last follow-up: 6 years, range: 2.8–23), while 4% were able to walk with support. Persistent motor symptoms, including movement disorders (75%), were present in most individuals. Hyperreflexia was reported in 68%, in most cases associated with spasticity (prevalence 49%), predominately in the lower limbs. Additional movement disorders included a hand tremor (30%), dystonia (35%), classified as focal in 18%, generalized in 14% and not further specified in 3%, as well as undefined dyskinesia of the limbs (30%). Stereotypical hand movements were rarely found (7%). In a subset, dysarthria (12%) was reported. Gait disturbances (56%), including an unsteady, spastic or crouched gait, were among the most commonly observed motor manifestations; in one case this led to secondary loss of ambulation. Gait examinations of patients with different degrees of gait impairment are provided in Supplementary Videos 2–4. Reduced muscle bulk with distal muscle atrophy was present in 26%. Infantile muscular hypotonia was reported in 21%, while overall hypotonia across all age groups was present in 42%, mostly reported as truncal hypotonia. Abnormal eye movements were reported in some individuals (21%), including external ophthalmoplegia (16%), oculomotor apraxia (4%) and rotational nystagmus (2%). Other ocular symptoms encompassed ptosis (35%) and strabismus (28%). Excessive drooling (21%) and swallowing dysfunction (26%) were relatively common, in all cases, except for one case without further information, requiring gastrostomy or nasogastric tube feeding.

**Figure 3:**
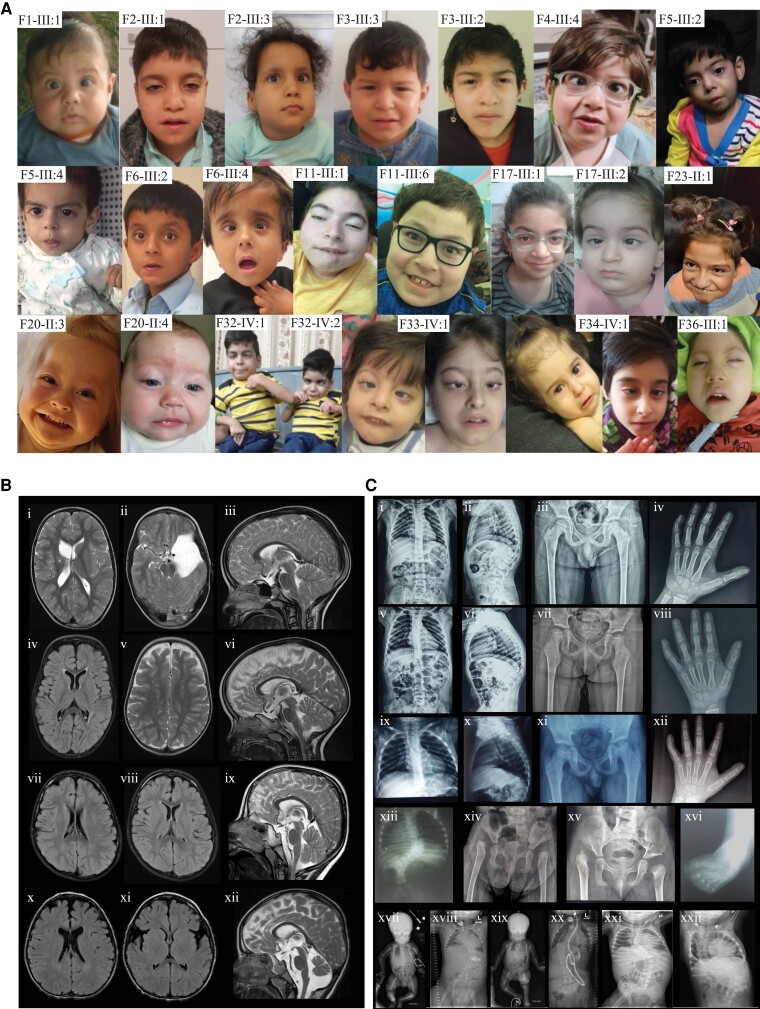
**Facial features, neuroimaging and skeletal radiographs**. (**A**) Facial photographs of individuals with biallelic *TOR1A* variants at different ages, showing the characteristic facial features, which include narrow forehead (bifrontal/bitemporal narrowing), broad/full nasal tip, full cheeks, thick/full vermilion of lower lip and broad, tall or pointed chin. For individuals F33-IV:1 and F34-IV:1, photographs at different ages are provided. (**B**) Neuroimaging findings in individuals with *TOR1A* variants. Findings for F2-III:1 (**i**–**iii**) include an arachnoid cyst in left middle and anterior cranial fossae (**ii**), areas of cystic encephalomalacia in the right basal ganglia with *ex-vacuo* dilation of the right lateral ventricular body (**i**), white matter volume loss (**i**) and Chiari malformation type 1 (**iii**). Findings for F2-III:3 (**iv**–**vi**) include foci of T_2_ hyperintense signal in the bilateral subcortical and periventricular white matter (**iv** and **v**), white matter volume loss (**iv**) and hypoplastic corpus callosum posterior body and isthmus (**vi**). Findings for F32-IV:1 (**vii**–**ix**) include small foci of fluid-attenuated inversion recovery hyperintense signal in the periventricular white matter (**vii** and **viii**), prominent posterior fossa cisterns and mega cisterna magna (**ix**). Findings for F29-II:2 (**x**–**xii**) include foci of T_2_ hyperintense signal in the bilateral subcortical and periventricular white matter (**x**), parenchymal volume loss of the frontal lobes with prominence of the sylvian fissures (**xi**), mildly hypoplastic corpus callosum, vermian hypoplasia, prominent posterior fossa cisterns and mega cisterna magna (**xii**). (**C**) Skeletal radiographs of individuals with *TOR1A* variants. Findings for F2-III:1 (**i**–**iv**) include mild scoliosis and thoracic kyphosis centred at the level of a hypoplastic T12 vertebra (**i** and **ii**), mild flattening of the capital femoral epiphyses (**iii**) and fifth finger clinodactyly (**iv**). Findings for F2-III:3 (**v**–**viii**) include mild scoliosis and thoracic kyphosis centred at the level of a hypoplastic T12 vertebra (**v** and **vi**), bilateral coxa valga with loss of height of the capital femoral epiphyses and slender femoral shafts (**vii**) and fifth finger clinodactyly (**viii**). Findings for F1-III:1 (**ix**–**xii**) include mild thoracic kyphosis (**ix** and **x**), mild flattening of the capital femoral epiphyses (**xi**) and fifth finger clinodactyly (**xii**). Findings for F3-III:2 (**xiii** and **xiv**) include 11 ossified ribs on the right and 12 on the left, thoracolumbar scoliosis (**xiii**) and dysplastic acetabula with dislocated capital femoral epiphyses and early formation of pseudo acetabula, absent ossification of both capital femoral epiphyses and short femoral necks (**xiv**). F3-III:3 (**xv**) had dysplastic acetabula, right hip dislocation with early formation of a pseudo acetabulum, absent and delayed ossification of the left capital femoral epiphysis, mild irregularity and sclerosis of the left acetabulum and short femoral necks. F33-IV:1 (**xvi**) had talipes equinovarus. Findings for F22-III:1 at birth (**xvii**) and 17 months of age (**xviii**). (**xvii**) Anteroposterior radiograph of the baby. The long tubular bones are slender and somewhat overmodelled. Allowing for patient positioning, there is no scoliosis and the hip joints are not dislocated. Umbilical vein catheter, endotracheal tube and nasogastric tube noted. (**xviii**) Anteroposterior radiograph of chest and abdomen. There are 12 pairs of ribs and normal segmentation. There is now a significant thoracolumbar scoliosis concave to the right. There is absent ossification of bilateral dislocated femoral heads, with hypoplastic acetabula and early pseudoacetabula formation. The long tubular bones are gracile. Tracheostomy and gastrostomy noted. Findings for F22-III:2 at birth (**xix**) and 2 years of age (**xx**). (**xix**) Anteroposterior radiograph of the baby. There are 12 pairs of ribs and normal segmentation. There is a thoracolumbar scoliosis concave to the left. The long tubular bones are slender and somewhat overmodelled. The hip joints do not appear dislocated. Umbilical vein catheter and nasogastric tube noted. (**xx**) Anteroposterior radiograph of chest and abdomen. The thoracolumbar scoliosis has progressed. There is absent ossification of bilateral dislocated femoral heads, with hypoplastic acetabula and pseudoacetabula formation. The long tubular bones are gracile and appear of reduced radiodensity. Tracheostomy, ventriculoperitoneal shunt and gastrostomy noted. Findings for F23-II:1 (**xxi** and **xxii**). Anteroposterior radiograph of chest and abdomen at 1 year 1 month (**xxi**) and 8 years 5 months (**xxii**) demonstrate a progressive and significant thoracolumbar scoliosis concave to the right. The earlier radiograph shows dislocation of the right hip joint; the left hip joint does not appear to be dislocated on the limited view available.

A detailed assessment of facial features in a subset of 18 individuals and six previously published cases with available photographs showed a broad range of dysmorphic signs (Fig. 3A and Supplementary material, File 4). The most prevalent included a broad/full nasal tip (95.8%), narrowing of the forehead (79.2%) and full cheeks (70.8%) (Fig. 3A). Further characteristic features encompassed highly arched eyebrows (37.5%), unilateral or bilateral ptosis (37.5%), thick/full vermilion of the lower lip (41.7%), a broad, tall or pointed chin (45.8%), a small, narrow mouth (29.2%) and retrognathia (29.2%).

Other clinical manifestations included umbilical or inguinal hernias (47%) and visual abnormalities (40%). In a small subset of individuals bladder and bowel function were impaired with persistent urinary or faecal incontinence (12%). Seizures and hearing impairment were less common (19% and 14%, respectively). Pain, a symptom that is frequently reported in AMC,^28^ was not systematically assessed in our cohort but reported in a subset of cases, in association with contractures (Supplementary material).

### Radiological assessment

Brain MRI studies were available for 11 individuals. Shared neuroimaging features included a hypoplastic corpus callosum (72%), foci of signal abnormality in the subcortical and periventricular white matter (55%), diffuse white matter volume loss (45%), mega cisterna magna (36%) and arachnoid cysts (27%) (Fig. 3B). Imaging findings present in single cases included Chiari malformation type 1 (F2-III:1), vermian hypoplasia, areas of cystic encephalomalacia in the basal ganglia (F2-III:1) and punctate parenchymal calcifications (F5-III:2). Case-based descriptions of the imaging findings are provided in the Supplementary material.

With respect to skeletal features, common radiographic findings included acetabular dysplasia with dislocated hips and scoliosis. Other nonspecific findings were slender bones, clinodactyly and kyphosis (Fig. 3C).

### Survival analysis

Overall survival was 71% (*n* = 37/52) at a median age at last follow-up of 3 years (range: 0–24). The two foetuses from Family 16, as well as the two cases reported by Gonsalves *et al.*^15^ (F38-II:3 and F38-II:4) and one of the siblings reported by Pehlivan *et al.*^11^ (F29-II:2) were excluded from survival analysis, since the affected pregnancies were medically terminated (in Families 16 and 29 due to FADS, in Family 38 due to multiple foetal anomalies). In the female population, 23% (5/22), including two previously reported individuals,^13,14^ were deceased at the time of reporting, with the oldest female being 23 years old. Of the males, 67% (*n* = 20/30) were alive, while 33% (*n* = 10/30) were deceased. The oldest male individual reported to date is 24 years old. Death occurred in early infancy, at a median age of 1.2 months (range 1 week–9 years) due to respiratory failure, cardiac arrest or sepsis.

### Molecular spectrum and analysis of genotype-phenotype correlations

**Figure 4:**
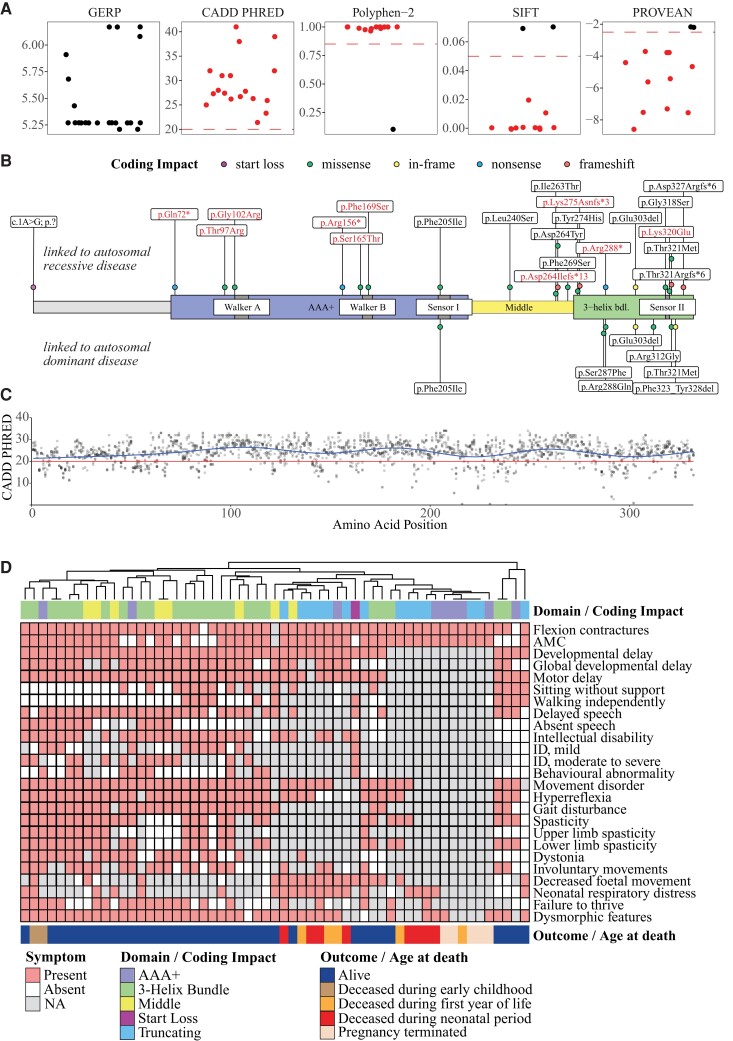
**Molecular characteristics**. (**A**) Variant effect prediction scores for all 22 distinct *TOR1A* variants. Genomic Evolutionary Rate Profiling (GERP) scores ranged from −12.3 to 6.17 show the level of conservation, with scores close to 6.17 indicating a high level of conservation. CADD Phred, Polyphen-2, SIFT and PROVEAN scores inferred the level of deleteriousness. Recommended thresholds are indicated with dashed red lines. Variants rated as likely to be deleterious are coloured in red. (**B**) Schematic of the Torsin-1A primary protein structure with its functional domains. Amino acid positions of variants linked to autosomal-recessive *TOR1A*-related disorders are plotted above and variants reported in autosomal-dominant DYT-*TOR1A* torsion dystonia below the Torsin-1A protein. Of note, in autosomal-dominant cases, only the p.Glu303del variant has been verified to cause classic early onset dystonia. The other variants reported for DYT-*TOR1*A have either been listed as pathogenic or likely pathogenic on ClinVar or have been reported in single cases. Variants printed in red text were likened to FADS or associated with early death. Coding impacts of variants are colour-coded. (**C**) CADD Phred scores for all possible missense variants were computed and mapped to the linear protein structure. A generalized additive model was used to predict the tolerance for genetic variation across the protein (blue line). The red line indicates the consensus cut off value for deleteriousness. (**D**) Exploration of genotype-phenotype correlations. Affected individuals were grouped based on the presence of core symptoms using a hierarchical clustering approach. The spectrum of core symptoms for each individual was visualized using a heatmap. Genetic information about the affected Torsin-1A domains (in case of missense or in-frame variants) or coding impacts of the individual *TOR1A* variants (in case of truncating or the start loss variant) was annotated above the heatmap. Domains included individuals with at least one missense or in-frame variant in the ‘3-Helix Bundle’ (*n* = 25), ‘AAA+’ (*n* = 9) and ‘Middle’ (*n* = 8) domain of Torsin-1A. Individuals with biallelic frameshift or nonsense variants were assigned to the ‘Truncating’ group (*n* = 14). The individual with the homozygous start loss variant was assessed separately. Due to the small number of cases within the AAA+ domain, further stratification into subdomains (‘Walker A’ or ‘Walker B’) was not done. Clinical outcomes were annotated blow the heatmap.

Consanguinity was reported in 50% of families. Biallelic variants were homozygous in 34 families and compound-heterozygous in six. Twenty-two distinct *TOR1A* variants were identified, including newly identified variants (*n* = 14) and those previously reported (*n* = 8). The variants were either absent or present, with a very low allele frequency across multiple variant frequency databases, and total allele counts ranged from 0 to 344 per 2 000 000. Details of the databases can be found in the Supplementary material. *In silico* analysis of the vast majority of these variants predicted high conservation of the affected amino acid residues and deleteriousness of the respective genomic changes (Fig. 4A and Supplementary material). The most frequent and only in-frame variant in our cohort was the recurrent three nucleotide deletion c.907_909delGAG (p.Glu303del), which in a heterozygous state is responsible for the majority of DYT-*TOR1A* cases.^5^ This variant was homozygous in eight families, followed by the truncating c.862C>T (p.Arg288*) variant, which occurred in a homozygous state in six families. Thirteen variants in our cohort were missense variants that affected single amino acid residues, predicted to retain residual protein function. Four distinct frameshift variants were identified, all of which were located in the last exon (5/5) of *TOR1A*, thus expected to result in protein truncation but unlikely to cause nonsense mediated decay (NMD). Thereby, p.Asp327Argfs*6 and p.Thr321Argfs*6 affected the C-terminal 6 and 12 amino acid residues, respectively, while the remaining two variants (p.Asp264Ilefs*13 and p.Lys275Asnfs*3) were predicted to lead to a loss of larger portions of the protein. Of the two nonsense variants, p.Arg288* was predicted to result in a truncated protein with loss of the last 45 amino acids, whereas p.Gln72* and p.Arg156* were expected to lead to NMD. Furthermore, one homozygous start loss variant (c.1A>G) with unknown consequence was present. This variant was classified as likely pathogenic based on *in silico* analyses (Supplementary material). Haplotype analysis of the exonic regions surrounding the p.Glu303del variant from four independent Iranian families revealed at least three distinct haplotypes, which indicates that this recurrent variant most likely arose independently even within the Iranian population (Supplementary Fig. 2).

Additional genetic testing to identify contributing causes for the patients’ phenotypes was performed in 19 individuals, including karyotyping (*n* = 13), chromosomal microarray analysis (CMA) (*n* = 12), mitochondrial DNA testing (*n* = 3) and in some cases targeted single gene testing for differential diagnoses of congenital arthrogryposis syndromes (*SMN1, RAPSN, CHRNG, GLRA1*). Chromosomal aberrations of unknown significance were found in two cases (duplications on chromosome 15q22.33q23 in Case F19-II:1 and on chromosome 1q21.1 in Case F10-II:1). Furthermore, in a few cases, panel and exome sequencing identified additional genetic variants (mostly benign or of unknown significance) in genes unrelated to the reported phenotypes. A detailed list of all genetic analyses performed, along with their results is provided in the Supplementary material.

The distribution of *TOR1A* variants found in biallelic *TOR1A*-related disorders as well as in the heterozygous state in DYT-*TOR1A* is shown in Fig. 4B. Most variants, including all variants reported in DYT-*TOR1A* (with the exception of p.Phe205Ile), clustered in the C-terminal region, mainly the 3-helix bundle domain of the Torsin-1A protein that has been shown to mediate interactions with torsin activator proteins.^24^ Seven variants (p.Gln72*, p.Thr97Arg, p.Gly102Arg, p.Arg156*, p.Ser165Thr, p.Phe169Ser and p.Phe205Ile) affected the AAA+ domain, containing the functionally important Walker A box and Walker B box domains. An overall low tolerance for genetic variation was suggested by a computational model of CADD Phred scores for all possible missense variants along the Torsin-1A structure (Fig. 4C).

To explore genotype-phenotype associations, individuals were categorized into phenotypic groups based on the presence of core clinical symptoms using a hierarchical clustering approach (Fig. 4D). For individuals carrying at least one missense or in-frame variant, suggesting residual protein function, genetic information was annotated by mapping the disease-associated *TOR1A* variants to the affected domains of the Torsin-1A protein using the categories ‘AAA+’, ‘Middle’ and ‘3-Helix Bundle’. In cases with biallelic frameshift or nonsense variants, the coding impact ‘Truncating’ was annotated. One individual carrying a start loss variant (c.1A>G) with unknown consequence was assessed separately. Two main groups were discernible based on hierarchical clustering: (Group 1) mostly individuals carrying at least one missense or in-frame variant in the C-terminal domain (3-helix bundle and middle domain); and (Group 2) predominantly individuals carrying biallelic truncating variants or biallelic missense variants in the Walker A/B domain. Individuals in Group 1 depicted a broad range of motor symptoms and cognitive abilities ranging from mild gait disturbance and normal cognition to severe arthrogryposis and GDD, and were alive within the observation period. Group 2 was associated with early death and particularly severe phenotypes including cases with FADS prompting early termination of pregnancy. Based on these findings, a closer analysis of variants associated to particularly severe presentations was conducted. Ten homozygous variants, affecting 20 individuals, were associated with early death in infancy or childhood (median age at death: 1.2 months, range: 1 week–9 years). This included five truncating variants, namely the p.Gln72*, p.Arg156* and p.Arg288* nonsense variants and the two frameshift variants p.Asp264Ilefs*13 and p.Lys275Asnfs*3, as well as five homozygous missense variants located in the AAA+ domain, p.Thr97Arg and p.Gly102Arg in close proximity to the Walker A motif, p.Ser165Thr and p.Phe169Ser linked to the Walker B motif, as well as the C-terminal p.Lys320Glu variant (Fig. 4B). The p.Gln72* nonsense variant (Family 16) and the previously reported p.Phe169Ser^11^ variant (Family 29) within the functionally important Walker B domain were associated with severe FADS, prompting early termination of pregnancy in three individuals (F16-II:1, F16-II:2 and F29-II:2), and a severe congenital arthrogryposis syndrome in two cases with death at the age of 1 week (F29-II:5) and 9 months (F29-II:1). Similarly, the previously reported p.Gly102Arg variant, located within the Walker A domain, was associated with multiple foetal anomalies which led to termination of two subsequent pregnancies in one family (Family 38).^15^

## Discussion

In this study, we report a cohort of 57 individuals with biallelic *TOR1A* variants, including 42 novel cases and all previously reported individuals. Through a systematic analysis, we delineate the clinical, molecular and imaging spectrum of this rare syndrome and identify possible genetic predictors for survival and disease severity. Our findings expand the clinical and molecular spectrum of *TOR1A*-related disorders, underscore the impact of loss of *TOR1A* function on early CNS development and provide a framework for future natural history studies and potential therapeutic trials.

Overall, the clinical spectrum in individuals with biallelic *TOR1A* variants is consistent with a syndrome of AMC along with an NDD with ID, pyramidal dysfunction and gait disturbances. The diagnosis of AMC is purely descriptive, as multiple factors that interfere with normal foetal movements can result in joint contractures.^29^ A substantial proportion of cases of FADS and AMC is due to pathogenic variants in primary neuromuscular genes,^30^ and it is intriguing in this context that Torsin-1A interacts both directly and indirectly with a number of nuclear envelope proteins, some of which have been implicated in both neuromuscular disorders and AMC.^31^ Additionally, genes associated with AMC which are linked to early onset movement disorders including dystonia have been reported, highlighting potential shared molecular pathways.^32,33^

While congenital or early contractures of multiple joints were present in most cases, the spectrum of neurological symptoms was variable and broad. Developmental delay encompassed delayed acquisition of motor milestones such as unsupported sitting and independent walking, as well as delayed speech and language development, with a subset of individuals remaining non-verbal. Intellectual abilities were impaired in most cases ranging from mild cognitive deficits to severe ID, while some individuals were reported with normal cognition. Persistent and progressive motor symptoms included multifactorial gait impairment, and while 4% were dependent on a walking aid, 34% of individuals never attained the ability to walk. Pyramidal dysfunction was common, and in most cases evolved to a spastic paraparesis. Additional movement disorders consisted of likely multifactorial gait disturbance, a hand tremor, and focal or generalized dystonia. The phenomenology of the tremor in our cohort was not systematically assessed but was reported as a dystonic tremor in two cases (F23-II:1 and F27-III:1), with partial improvement after levodopa treatment in one case (F23-II:1). No differences in the frequency of phenotypic features were found between females and males. Regarding the evolution of the disease over time, the age span and follow-up period of our cohort is currently too small to draw definitive conclusions. In single cases, motor symptoms, particularly contractures, improved, in line with a previous report that suggests dynamic changes with improvement of contractures and tremor, while strabismus was reported to worsen over time.^9^ Longitudinal follow-up studies will be necessary to evaluate these observations. Characteristic facial features assessed in a subset of individuals included ptosis, a broad/full nasal tip, narrowing of the forehead and full cheeks. Shared neuroimaging abnormalities included a hypoplastic corpus callosum, diffuse white matter volume loss, foci of signal abnormalities in the subcortical and periventricular white matter and arachnoid cysts.

Taking all reported cases with biallelic *TOR1A* variants into account, survival was approximately 71% at a median age at last follow-up of 3 years (range: 0–24). Most female individuals were alive at the time of publication, with the oldest having reached early adulthood. Five female individuals passed away (age 1 week–4 months), due to respiratory complications or without a reported cause of death. In the male population, about a third passed away early, at a median age of 3.5 months (range: 1 week–9 years). Respiratory failure, cardiac arrest and sepsis were reported as causes of death. The oldest male individual reported is currently 24 years old. The small sample size and heterogenous nature of our cohort preclude attribution of sex-specific factors to the higher mortality in males, but this should be explored in larger and longitudinal studies.

Our study has several limitations: (i) The cross-sectional data, limited age range and small subset of individuals with longitudinal data render a comprehensive assessment of the natural history of this ultra-rare condition impossible. (ii) Given the interdisciplinary and multicentre design across different health care settings, phenotyping of affected individuals was subject to ascertainment bias and missing data. Therefore, the true frequencies of clinical features associated with biallelic *TOR1A* variants might be underestimated. This seems particularly important for clinical manifestations beyond early childhood, as the number of individuals in the adolescent or adult age group was limited. To facilitate phenotypic characterization of individuals with biallelic variants in *TOR1A*, we used HPO terminology to describe phenotypic features. Larger longitudinal studies will be needed to delineate the natural history.

Understanding of Torsin-1A function at the molecular level has provided insights into potential disease mechanisms in *TOR1A*-associated disorders. High metabolic turnover in post-mitotic tissues requires an intricate machinery of intracellular trafficking pathways and mechanisms that regulate protein homeostasis and quality control. Proteolytic machineries contain multi-subunit protease complexes and AAA-ATPases (ATPases associated with diverse cellular activities). Torsin-1A is a member of the AAA+ family of ATPases that assemble into multimers and derive energy from ATP hydrolysis to mediate conformational changes on substrate proteins.^34^ Torsin-1A localizes to the endoplasmic reticulum and nuclear envelope.^35^ Roles in protein quality control, vesicular trafficking, lipid metabolism, cellular architecture and nuclear envelope organization have been described.^36,37^ Unlike other members of the AAA+ superfamily of ATPases, Torsin-1A lacks intrinsic ATPase activity and requires binding to the Torsin-activator proteins LAP1 and LULL1.^38,39^ Torsin-1A has been predicted to form homohexamers that interact with LAP1 and LULL1, triggering ATP hydrolysis and disassembly.^40^

The majority of pathogenic *TOR1A* variants in both heterozygous and biallelic states were missense variants clustering in the C-terminal domain, rather than being uniformly distributed along the Torsin-1A protein. This suggests that disruption of functional domains rather than a complete loss of protein function might drive *TOR1A* pathology. In heterozygotes, the absence of pathogenic truncating variants argues for a dominant-negative effect rather than haploinsufficiency, corroborated by reports of asymptomatic carriers of heterozygous truncating variants in *TOR1A*^41^ and supported by gnomAD data.^42^ Indeed, functional studies have shown that the recurrent p.Glu303del variant, responsible for the majority of early-onset isolated dystonia (DYT-*TOR1A*) cases, exerted dominant-negative effects on wild-type Torsin-1A by destabilizing Torsin-1A binding to its activator proteins.^43,44^ Interestingly, heterozygous parents and siblings in our cohort were reportedly unaffected, except for single cases with leg muscle cramps (maternal parents F7-II:1 and F11-II:1), lower limb pain and stiffness (paternal parent F24-III:1) and possible restless leg syndrome and writer’s cramp (paternal parent F11-II:2, heterozygous sibling F11-III:4). Of note, information on heterozygous parents and siblings was purely based on family histories; no reports on systematic clinical examination were available for these individuals. Findings are overall consistent with the reduced penetrance of around 30% for individuals carrying the heterozygous p.Glu303del variant.^6^ In addition, most of the parents were relatively young and might thus develop symptoms in the future.

With biallelic variants, several patterns became apparent: first, almost all individuals that were alive at last follow-up carried variants affecting the second half of the Torsin-1A protein (except one individual with a homozygous stop loss variant). Similarly, all individuals reported to have normal intellectual abilities were part of this cluster. Biallelic truncating variants were not present in this subgroup, suggesting, that one allele of a C-terminal in-frame or missense variant might be sufficient to lead to an attenuated phenotype.

By contrast, our data support the hypothesis that biallelic truncating variants might be associated with early postnatal death, in agreement with available *Tor1A* mouse models.^45,46^ We identified eight individuals from six families harbouring the recurrent p.Arg288* nonsense variant in a homozygous state, as well as five additional individuals carrying the homozygous p.Gln72* (*n* = 2), p.Arg156*, p.Asp264Ilefs*13 and p.Lys275Asnfs*3 variants, respectively. Though only two of these variants were expected to lead to NMD, all affected children, except for one, died in the pre-/neonatal period (*n* = 9) or within the first 6 months of life (*n* = 3). F27-III:1, carrying the homozygous p.Arg288* variant was, at last follow-up at 2 months of age, under palliative care. Interestingly, two individuals in our cohort carrying the p.Arg288* variant *in trans* with the recurrent p.Glu303del in-frame variant (F23-II:1 and F10-II:1) were alive at last follow-up at 8 and 24 (the oldest individual in our cohort) years of age, respectively, underscoring the hypothesis that one non-truncating allele might be sufficient to prevent early death.

The next pattern that became visible was the association of missense variants affecting specific functional protein domains with severe disease and early death. Eight individuals were identified carrying variants within or in close proximity to the functionally important Walker A (p.Thr97Arg, p.Gly102Arg) and Walker B (p.Ser165Thr and p.Phe169Ser) motifs that are important for nucleotide binding and hydrolysis.^36^ All of these variants were associated with severe congenital arthrogryposis and early death at a median age of 9 months (range: 1 week–9 years).

Finally, a few variants were associated with an exceptionally severe phenotype and early termination of pregnancy. Two homozygous variants (p.Gln72* and p.Phe169Ser) were identified that led to FADS. The p.Gln72* variant segregated in a family with two healthy heterozygous parents, who had two subsequent terminated pregnancies at 25 weeks of gestation due to absent foetal movements and oligohydramnios. Post-mortem examination showed severe generalized arthrogryposis in both foetuses. Exome sequencing identified the truncating p.Glu72* variant in a homozygous state. Similarly, the p.Phe169Ser missense variant, linked to the Walker B motif, occurred in a family with one case of FADS (pregnancy terminated) and two children that had severe congenital arthrogryposis and died at the ages of 1 week and 9 months. These findings suggest particularly severe disease associated with *N*-terminal truncating variants as well as missense variants associated with functionally important domains. Along these lines, two foetuses from a previously reported family harbouring the p.Gly102Arg variant, located within the Walker A domain, were, though not explicitly linked to FADS, reported to have multiple foetal anomalies which led to termination of both pregnancies.^15^ Regarding the start loss variant (c.1A>G, p.?), we are currently unable to predict functional consequences on protein function. Although the affected individual (F21-III:1) shows comparably mild symptoms, the clinical course is consistent with *TOR1A*-related disorders, supported by *in silico* analyses of the start loss variant predicting deleteriousness. An exceptional case is presented by a previously reported individual harbouring the homozygous missense variant p.Lys320Glu. Although located at the C-terminal end of the Torsin-1A protein, in close proximity to variants associated with milder disease, this patient showed severe congenital arthrogryposis, postnatal respiratory distress, seizures, blindness, deafness and cerebral MRI abnormalities and died at 3 months of age. No additional genetic aetiology was identified on exome sequencing and chromosomal microarray analysis.^16^

Together, these findings suggest that variants depending on their coding impact, position along the Torsin-1A protein, as well as proximity to functional important domains, might be implicated in the phenotypic variability observed in *TOR1A-*related disorders. Larger cohorts are however needed to conduct reliable subgroup analyses. This may allow for more precise genotype-phenotype correlation analyses and stratification of outcomes.

In conclusion, autosomal-recessive *TOR1A*-related disorders span a wide phenotypic spectrum, ranging from mild motor symptoms to severe arthrogryposis, global developmental delay and early death. Careful assessment of phenotype-genotype correlations offers a framework for patient counseling and stratification for future studies.

## Supplementary Material

awad039_Supplementary_Data
