# Supplementary material for: The clinical and genetic spectrum of autosomal-recessive TOR1A-related disorders
Source: Brain. 2023 Feb 9;146(8):3273–88. doi: 10.1093/brain/awad039 (PMC10393417; doi:10.1093/brain/awad039)
Supplement: awad039_Supplementary_Data [file awad039_supplementary_data.zip › brain-2022-01664-File007.pdf]

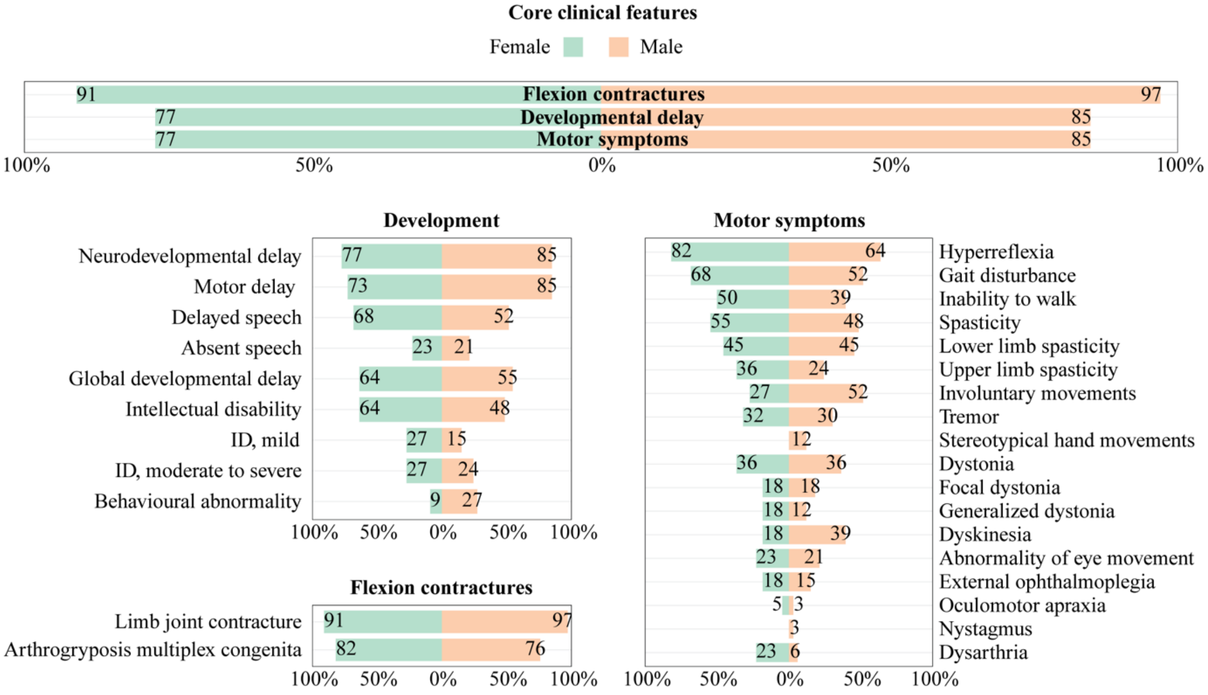

Supplementary Figure 1 Clinical spectrum by sex. (A) A total of 22 females and 33 males were examined (two individuals were excluded since information on sex was not available). Frequencies of core clinical features were present in the majority of individuals in our cohort. Below, a detailed breakdown of HPO derived phenotypic features for the core symptom categories “Development”, “Flexion contractures” and “Motor symptoms” is shown. Frequencies were printed on the respective bars.

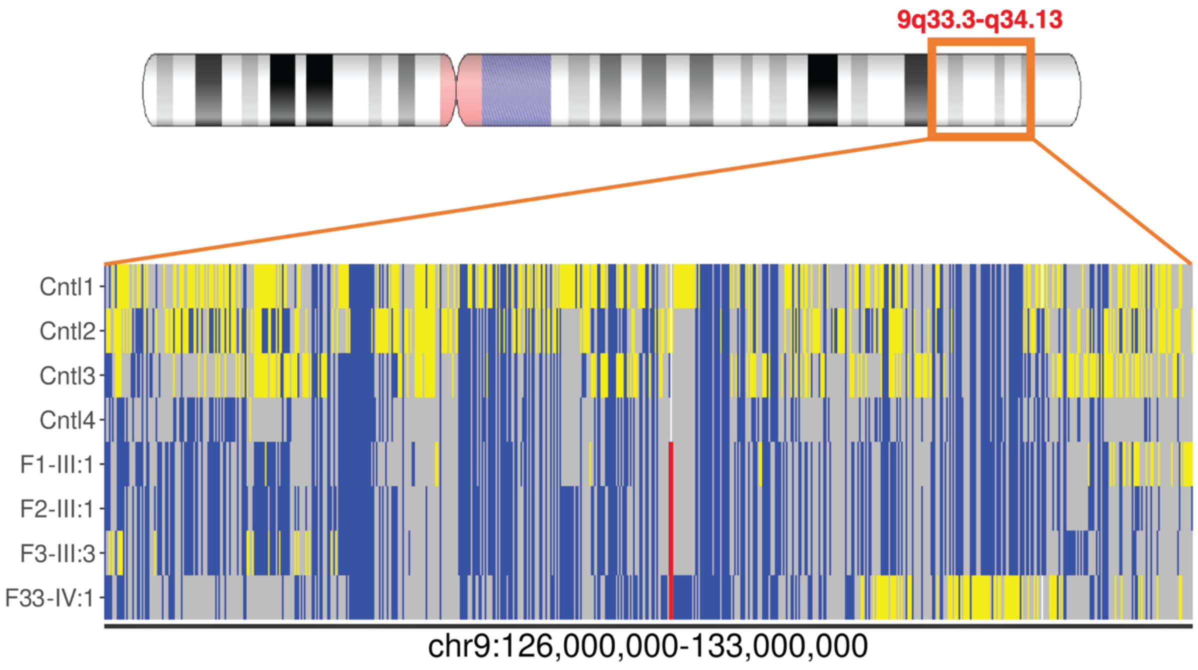

Supplementary Figure 2 Haplotype analysis of the recurrent p.Glu303del variant. Pictorial representation of exome genotype data from 4 affected individuals across ~7 Mb of chromosome 9q33.3-q34.13 encompassing deletion of c.907_909delGAG in TOR1A (indicated by the red bar). Color codes are as follows: the pathogenic variant as red, homozygous variants as blue, heterozygous variants as yellow, and wild type as grey.
