# Supplementary material for: The clinical and genetic spectrum of autosomal-recessive TOR1A-related disorders
Source: Brain. 2023 Feb 9;146(8):3273–88. doi: 10.1093/brain/awad039 (PMC10393417; doi:10.1093/brain/awad039)
Supplement: awad039_Supplementary_Data [file awad039_supplementary_data.zip › brain-2022-01664-File008.pdf]

Supplementary File 1 Summary and characterization of variants identified in our study.

Information on genetic testing, variant characterization, allele frequencies in multiple databases and *in silico* predictions are provided.

Supplementary File 2 Summary of the phenotypic spectrum of *TOR1A*-related disorders.

Frequencies for all extracted phenotypic features for the total study population as well female and male subgroups are provided.

Supplementary File 3 Patient-based data.

Detailed information is provided for all individuals included in this study.

Supplementary File 4 Dysmorphology assessment.

Detailed descriptions of facial features for 24 patients.

Supplementary File 5 Neuroradiology assessment.

Detailed descriptions of neuroradiologic findings in 11 patients.

Video files:

Video 1: 8-month old female with a homozygous p.Gly318Ser variant in *TOR1A* with arthrogryposis multiplex congenita.

Video 2: 8-year-old male with a homozygous p.Glu303del variant in *TOR1A* and mild gait impairment.

Video 3: 7-year-old male with a homozygous p.Glu303del variant in *TOR1A* and moderate gait impairment.

Video 4: 13-year-old female with a homozygous p.Glu303del variant in *TOR1A* and severe gait impairment.
