# Supplementary material for: The clinical and genetic spectrum of autosomal-recessive TOR1A-related disorders
Source: Brain. 2023 Feb 9;146(8):3273–88. doi: 10.1093/brain/awad039 (PMC10393417; doi:10.1093/brain/awad039)
Supplement: awad039_Supplementary_Data [file awad039_supplementary_data.zip › brain-2022-01664-File010.pdf]

AMC5-*TOR1A* Neuroimaging Descriptions

<u>F1-III:1:</u>

Only low-resolution photos available. Foci of T2 hyperintense signal in the bilateral subcortical and periventricular white matter. Mildly hypoplastic corpus callosum.

<u>F2-III:1:</u>

Prominent arachnoid cyst in left middle cranial fossa and extending into the sylvian fissure and anterior-inferior aspect of the left anterior cranial fossa. Associated mass effect with flattening of the left temporal pole and left cerebral peduncle, frontal and temporal sulcal effacement, widening of the sylvian fissure, and minimal left-to-tight midline shift. Areas of cystic encephalomalacia with surrounding gliosis in the right caudate nucleus, putamen, and globus pallidus. Associated mild ex-vacuo dilation of the right lateral ventricular body. White matter volume loss. Hypoplastic anterior commissure. Caudal descent of the cerebellar tonsils 5 mm below the level of the foramen magnum, consistent with Chiari malformation type 1. Retroflexion of the odontoid process and Chiari malformation cause crowding of the foramen magnum.

<u>F2-III:3:</u>

Diffuse white matter volume loss with secondary prominence of the ventricles and sulci. Foci of T2 hyperintense signal in the bilateral subcortical and periventricular white matter. Hypoplastic corpus callosum posterior body and isthmus. Mildly prominent tortuous veins overlying the bilateral cerebral convexities.

<u>F3-III:3:</u>

Only low-resolution photos available. Subtle foci of T2 hyperintense signal in the bilateral subcortical and periventricular white matter. Mildly hypoplastic corpus callosum isthmus.

<u>F4-III:4:</u>

Only low-resolution photos available. Small arachnoid cyst in the left middle cranial fossa with no significant local mass effect. Diffuse white matter volume loss with secondary prominence of the ventricles and sulci. Hypoplastic corpus callosum. Mega cisterna magna.

<u>F5-III:2:</u>

Only low-resolution photos available. Arachnoid cyst posterior to the vermis with mild local mass effect and scalloping of the overlying occipital bone. Punctate calcifications in the right centrum semiovale, right lentiform nucleus, and right cerebellar hemisphere.

<u>F22-III:1:</u>

Only low-resolution photos available. Mildly hypoplastic corpus callosum. Mildly prominent superior cerebellar cistern.

F26-II:1:

Only low-resolution photos available. Mildly hypoplastic corpus callosum. Mildly prominent subarachnoid spaces overlying cerebral convexities. Mega cisterna magna. Retroflexion of the odontoid process.

F32-IV:2:

Mildly hypoplastic corpus callosum. Foci of T2 hyperintense signal in the bilateral subcortical and periventricular white matter. Parenchymal volume loss of the frontal lobes with prominence of the sylvian fissures. Vermian hypoplasia with secondary widening of the foramen of Magendie. Prominent posterior fossa cisterns. Mega cisterna magna. Retrognathia. Mildly prominent adenoids.

F33-IV:1:

Small foci of FLAIR hyperintense signal in the periventricular white matter. Prominent posterior fossa cisterns. Mega cisterna magna.

F34-IV:1:

Only low-resolution photos available. Mildly hypoplastic corpus callosum. Diffuse white matter volume loss with secondary prominence of the ventricles and sulci. Foci of T2 hyperintense signal in the bilateral subcortical and periventricular white matter.
